# Verses

**Published:** 1906-05

**Authors:** 


### VERSES.

### A Week Off.

Drop a week from money-getting, let old
Duty take a rest; try what makes life worth
the living, see what gives to work its zest.

*Alk. Clinic.*

---

### Your Subscription.

How dear to our heart
Is the price of subscription,
When honest subscribers
Present it to view.
Of him who'll not pay up,
We shrink from description,
For perchance, dear reader,
That one might be YOU.

*Exchange.*

---

### Human Nature.

We have a stack of trouble in
This little vale of tears;
The world is full of grief and sin,
The world is full of fears.
A man finds endless cares are his
As through this life we go,
And of them all the greatest is
Imaginary woe.

We struggle bravely under debt,
We struggle under pain.
And strive the upper hand to get
The victory of gain.
Though endless troubles by us whiz
We ward off every blow—
The only thing that floors us
Imaginary woe.

*Exchange.*

---

### My Dog Is My Friend.

Sum say dey's frens, but few am true;
Hit's esy ter talk as fallin fum er log,
But of all dem frens that is true blue,
Dere's nun so good es myr yaller possum dog.

I mer drive 'im fum de homny pot,
An run 'im roun' wid er hickory stick,
Keep 'im out doors, wedder cole or hot,
He's reddy ebry time mer han' fer ter lick.

He'll bark and frisk wen I am glad,
An lick de skillet ter de bery dregs,
But ef I'm down, en mer luck am sad
He'll mope all erbout wid tail twixt his legs.

So gib me yer paw, mer allus true fren,
I wish fer sunshine and small 'mount ob fog,
Em de ways ob life up ter de end,
Fer you an me, mer leetle possum dog.

*B. H. Teague.*

